# Carbon Nanotubes Incorporated Z-Scheme Assembly of AgBr/TiO_2_ for Photocatalytic Hydrogen Production under Visible Light Irradiations

**DOI:** 10.3390/nano9121767

**Published:** 2019-12-11

**Authors:** Nasir Shezad, Ibrahim M. Maafa, Khairiraihanna Johari, Ainy Hafeez, Parveen Akhter, Maira Shabir, Ali Raza, Hirra Anjum, Murid Hussain, Muhammad Tahir

**Affiliations:** 1 Department of Chemical Engineering, Faculty of Engineering, Universiti Teknologi PETRONAS, Bandar Seri Iskandar 32610, Perak, Malaysia; nasir.shehzad@cuilahore.edu.pk (N.S.); khairiraihana.j@utp.edu.my (K.J.); hirraanjum@yahoo.com (H.A.); 2 Department of Chemical Engineering, COMSATS University Islamabad, Lahore Campus, Defence Road, Off Raiwind Road, Lahore 54000, Pakistan; ainyhafeez@cuilahore.edu.pk (A.H.); mairashabir@gmail.com (M.S.); engineeraliciit@gmail.com (A.R.); 3 Department of Chemical Engineering, College of Engineering, Jazan University, Jazan 45142, Saudi Arabia; 4 Centre of Contaminant Control & Utilization, Institute of Contaminant Management (ICM), Universiti Teknologi PETRONAS, Bandar Seri Iskandar 32610, Perak, Malaysia; 5 Department of Chemistry, The University of Lahore, 1-km Raiwind Road, Lahore 54000, Pakistan; parveen.akhter@chem.uol.edu.pk; 6 Chemical Reaction Engineering Group (CREG), School of Chemical and Energy Engineering, Universiti Teknologi Malaysia, Skudai, Johor Bahru 81310 UTM, Johor, Malaysia

**Keywords:** Z-scheme photocatalyst, solid-state electron mediator, carbon nanotubes, titanium dioxide, silver bromide, hydrogen production

## Abstract

Photocatalytic H_2_ production is a promising strategy toward green energy and alternative to carbon-based fuels which are the root cause of global warming and pollution. In this study, carbon nanotubes (CNTs) incorporated Z-scheme assembly of AgBr/TiO_2_ was developed for photocatalytic H_2_ production under visible light irradiations. Synthesized photocatalysts were characterized through transmission electron microscope (TEM), X-ray photoelectron spectra (XPS), X-ray diffractometer (XRD), Fourier transform infrared (FTIR), photoluminescence spectra (PL), Brunauer Emmet-Teller(BET), and UV-vis spectroscopy analysis techniques. The composite photocatalysts exhibited a H_2_ production of 477 ppm which was three-folds higher than that produced by TiO_2_. The good performance was attributed to the strong interaction of three components and the reduced charge recombination, which was 89 and 56.3 times lower than the TiO_2_ and AgBr/TiO_2_. Furthermore, the role of surface acidic and basic groups was assessed and the photocatalytic results demonstrated the importance of surface functional groups. In addition, the composites exhibited stability and reusability for five consecutive cycles of reaction. Thus, improved performance of the photocatalyst was credited to the CNTs as an electron mediator, surface functional groups, higher surface area, enhanced charge separation and extended visible light absorption edge. This work provides new development of Z-scheme photocatalysts for sustainable H_2_ production.

## 1. Introduction

Photocatalytic H_2_ production is an attractive approach to satisfy the worldwide energy requirements and to reduce the global warming. However, development of efficient photocatalyst with the ability to function under visible light irradiations and good charge separation is still a great challenge. State-of-the art Z-scheme photocatalysts offer a two-fold advantage of efficient charge separation and visible light solar energy utilization [1,2]. In Z-scheme photocatalytic system, compatible semiconductors with respect to their band positions establish efficient charge transfer mechanism which lessen the charge recombination. Further, the efficiency of charge separation is even higher in indirect Z scheme structure owing to the presence of electron mediators. The electron mediator expedites the process of charge transfer and reduce the recombination of electrons-holes pairs. These parted electrons and holes with high redox potential increase the efficiency of photocatalytic production [2,3].

The Z-scheme assembly of TiO_2_ with compatible semiconductors is considered as a promising strategy to enhance the charge transfer and light absorption capability [4,5]. Silver bromide (AgBr) with an indirect bandgap energy of 2.6 eV have hardly been used in photocatalysis because of decomposition problem under light irradiations. However, the coupling of AgBr with suitable semiconductors like TiO_2_ enhances the stability by developing heterojunction which fastens the transport of electrons [6] and prevent the degradation. The conduction band potential of AgBr is −0.30 eV allowing it to develop Z-scheme assembly with TiO_2_ whose conduction band is −0.5 eV [7]. Asi et al. [8] studied the AgBr/TiO_2_ photocatalysts for CO_2_ conversion into CH_3_OH and reported the stable activity for continuous four cycles. Similarly, AgBr particles supported over amine functionalized rGO were reported by Shah et al. [6] for the decomposition of methylene blue and they noticed stable activity for continuous 12 cycles. Further, electron mediator plays an important role in the efficiency of indirect Z-scheme system. Noble metals such as Ag, Au, and Pt have been reported as electron mediators for Z-scheme photocatalysts [9,10,11]. However, noble metals-based Z-scheme systems are costly and require complex synthesis protocols.

Nowadays, there is an increasing trend to develop carbon-based electron mediators such as graphene and carbon nanotubes owing to their high electrical conductivity [12,13,14]. Numerous studies have reported the use of graphene as an electron mediator because of high charge carrier separation capability. Similarly, carbon nanotubes have been used as an electron mediator owing to extraordinary electrical, optical, and physical properties. Recently, Mohamad et al. [13] have reported the Z-scheme assembly of g-C_3_N_4_/CNTs/TiO_2_ composites for the photocatalytic degradation of dyes. The composite exhibited almost six times higher degradation of phenol because of intimate surface contact between the two semiconductor through CNTs which reduced the charge recombination. Similarly, Boon-Joon et al. [3] reported CNTs as an electron mediator in Zn_0.5_Cd_0.5_S-MWCNT-TiO_2_ nanocomposites for H_2_ production. The CNTs efficiently transported the electrons from TiO_2_ to Zn_0.5_Cd_0.5_S. Yet, there are few studies which focus on carbon nanotubes as an electron mediator. Further, effective bonding is essential to achieve the desired charge separation and transportation through an electron mediator. The strength of bonding depends on the surface functional groups that promote the interaction between semiconductors. The surface functional groups play a dynamic role in enhancing the interaction of reactants with surface of photocatalyst and photocatalytic stability. For example, Nasir et al. [5] reported that AgBr/rGO/TiO_2_ exhibited higher efficiency in the basic medium rather than acidic. Acidic and basic groups can be introduced on the surface of the composite by functionalization. Additionally, the difficulty of pH control during the photoreaction experiment could also be avoided by functionalization of materials with different solvents which would be an added advantage to the stability of photocatalyst. However, there is limited study on coupling of CNTs as an electron mediator and the role of the functional groups in Z-scheme structures.

In this study, carbon nanotubes-incorporated Z-scheme assembly of AgBr/TiO_2_ was studied. The AgBr/bCNTs/TiO_2_ photocatalysts were synthesized by deposition of AgBr and TiO_2_ on CNTs followed by reflux treatment to develop effective interfacial bonding. Obtained photocatalysts were calcined and analyzed through several characterization techniques including TEM, XRD, XPS, Raman, PL, UV-vis spectroscopy, N_2_ adsorption and desorption technique. Performance of the AgBr/CNTs/TiO_2_ was studied for H_2_ production under visible light irradiations. Furthermore, role of functional groups was assessed by treating the CNTs with NaOH and NaOCl, and with H_2_SO_4_, HNO_3_ and CH_3_COOH solvents and integrated with AgBr/TiO_2_. In the end, the mechanism of photocatalytic H_2_ generation was proposed.

## 2. Experimental Section

### 2.1. Materials

Multi-wall carbon nanotubes (length 5 µm, Diameter 20 nm) were obtained from Carbon Nano-material Technology Co., Ltd. Pohang, South Korea. The cetyltrimethylammonium bromide (CTAB, C_19_H_42_BrN, ≥98%) and anatase TiO_2_ (99.50%) were obtained from Sigma-Aldrich, St. Louis, MO, USA. Hydrochloric acid (HCl, 37%), sodium hydroxide (NaOH) and sodium oxychloride (NaOCl) were obtained from R&M chemicals, Essex, U.K. Silver nitrate (AgNO_3_ 99%) and ethanol (C_2_H_5_OH, 99.99%) were obtained from Merck, KGaA, Darmstadt, Germany.

### 2.2. Preparation of Functionalized Carbon Nanotubes

The carbon nanotubes (CNTs) were dispersed into 1M NaOH and were boiled at 120 °C for 15 min. After that 30% NaOCl was added into dispersion followed by boiling for 2 h. The suspension was then cooled down to room temperature, filtered and neutralized by dispersing into the HCl solution. Obtained CNTs were washed with 0.1 M HCl and water to remove residual acid and base contents, followed by drying at 80 °C for 8 h. The product was denoted as bCNTs. Similarly, CNTs functionalization was carried out in acidic media containing 0.1 M H_2_SO_4_, 0.1 M HNO_3_, and 0.1 M CH_3_COOH at 120 °C for 4 h. The mixture was then filtered and washed several times with water to remove residual acid contents and denoted as aCNTs.

### 2.3. Preparation of AgBr/CNTs/TiO_2_

The AgBr/bCNTs/TiO_2_ was prepared by employing a two-steps approach which includes growth of AgBr over CNTs and bonding with TiO_2_ particles followed by reflux. A particular amount of bCNTs were distributed in absolute ethanol with the help of sonication for 1 h. The desired quantity of CTAB was added to the bCNTs mixture. The required weight of AgNO_3_ was dissolved in NH_3_ solution and mixed into the above suspension. The obtained mixture was stirred for 1 h for the uniform deposition of AgBr nanoparticles on the surface of bCNTs. Simultaneously, the desired quantity of TiO_2_ was distributed in absolute ethanol, sonicated and stirred for 1 h. The TiO_2_ suspension was mixed with AgBr/bCNTs solution and the resulting mixture was shifted to round neck flask and refluxed for 2 h at 80 °C to strengthen the interfacial bonding among AgBr, bCNTs and TiO_2_. The obtained product was filtered, dried in an oven at 100 °C and calcined at 300 °C for 2 h. A similar method was repeated to synthesize the AgBr/aCNTs/TiO_2_, where AgBr/TiO_2_ was synthesized using the same approach without the addition of the CNTs. Likewise, aCNTs/TiO_2_ and bCNTs/TiO_2_ were also prepared without the addition of AgNO_3_ and CTAB.

### 2.4. Characterizations of Photocatalyst

The surface morphology of the synthesized photocatalysts was studied employing a transmission electron microscope (TEM HITACHI HT7700, Tokyo, Japan) on different magnifications. Crystalline structure photocatalysts were investigated using an X-ray diffractometer (XRD, PANalytical X’pert^3^ powder, Malvern WR14 1XZ, U.K) with a diffraction angle range of 5° to 90° employing Cu α radiations (40 kV and 30 mA) at a scanning rate of 0.07878°. The surface chemical composition was investigated using X-ray photoelectron spectra (XPS) (Thermo scientific K-alpha (Kα) spectrometer, East Grinstead, U.K). The XPS spectrometer was equipped with Al Kα radiations with corrected calibration binding energies against the C_1_ of carbon fixed at 284.6 eV. Raman microscope (Lab RAM HR Evolution, HORIBA, Kyoto, Japan) with laser excitation at 325 nm in the range of 100–1000 cm^−1^ was used to record the PL spectra. The functional groups present on the surface of different photocatalysts were observed using Fourier transform infrared (FTIR) spectroscopy (Thermo Nicolet FTIR 6700, Thermo Scientific, Waltham, MA, USA) with wavelengths of 600 to 4000 cm^−1^. Optical properties of photocatalysts were studied using UV-visible spectrometer (Agilent Cary technologies 100 UV-vis spectrometer Model G9821A, Santa Clara, CA USA) in the range of 200 to 800 nm. Bandgap energies of photocatalysts were estimated from Tauc plots of Kubelka–Munk Function versus photon energy.

### 2.5. Photocatalytic H_2_ Production

The performance of the photocatalysts was evaluated for H_2_ generation from water using a slurry reactor as reported in our previous study [5]. The glass photoreactor consisted of two chambers with 35 W HID Xe lamp (intensity 20 mWcm^−2^, Philips, Selangor, Malaysia) as a source of light. Methanol was added as a sacrificial agent. Typically, 100 mg of photocatalyst was dispersed into a 20% methanol solution. Photoreactor setup was positioned on a magnetic stirrer for good dispersion of photocatalyst in the slurry. Prior to photoreaction, N_2_ gas was purged for 1 h to remove gaseous impurities from the reactor and also used as a carrier gas at the flow rate of 20 mL/min. Photoreaction was triggered by switching on Xe lamp. Gaseous samples were collected after an interval of 1 h in gas collecting bags and H_2_ was detected using Intelligent H_2_ analyser (BROTIE Technology Co., Ltd., Haidian District, Beijing China).

## 3. Results and Discussions

### 3.1. Characterization of Photocatalysts

**Figure 1:**
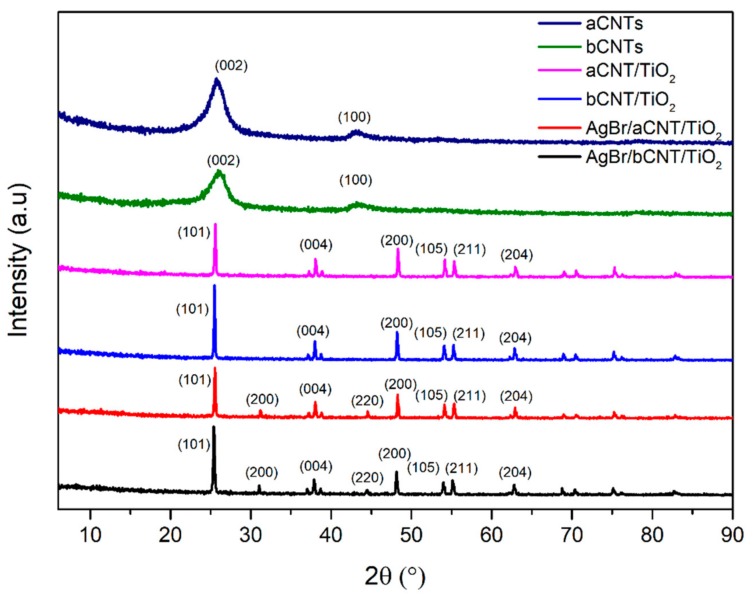
X-ray diffractometer (XRD) spectra of aCNTs, bCNTS, aCNTs/TiO_2_, bCNTs/TiO_2_, AgBr/aCNTs/TiO_2_ and AgBr/bCNTs/TiO_2_ photocatalysts.

Crystallinity and phase structures of aCNTs, bCNTs, aCNTs/TiO_2_, bCNTs/TiO_2_, AgBr/aCNTs/TiO_2_ and AgBr/bCNTs/TiO_2_ photocatalysts were studied using XRD analysis as shown in Figure 1. Debye–Scherrer equation and Bragg’s Law were employed to measure the particles size and interlayer spacing. Diffractogram of aCNTs and bCNTs have peaks at 26.2° and 45.68° corresponding to the (002) and (100) planes respectively. In aCNTs/TiO_2_ and bCNTs/TiO_2_ spectrum, peaks at 25.33°, 37.83°, 48.07°, 53.9°, 55.08°, 62.70°, 68.79°, 70.31° and 75.04° indicated the (101), (004), (200), (105), (211), (204), (116) and (220) crystalline planes of TiO_2_ (JCPDS-98-008-2084) [15]. The particles size was 44.79 nm measured from the prominent peak at 25.33°. The pattern of both aCNTs/TiO_2_ and bCNTs/TiO_2_ did not show any peaks for CNTs owing to the very low quantity of CNTs. In AgBr/aCNTs/TiO_2_ and AgBr/bCNTs/TiO_2_ spectra, there were additional new peaks at 31.21° and 44.54° representing the AgBr nanoparticles with face-centred cubic structure with crystal planes of (200) and (220) crystal plane, respectively [16]. No peak was observed for silver (Ag°) indicating the formation of only the AgBr/bCNTs/TiO_2_ photocatalyst_._ Similarly, no peak was found for CNTs because of marginal quantity, even dispersion and shielding effect of TiO_2_ peak at 2θ = 25.33° [17]. These results showed the successful formation of AgBr/aCNTs/TiO_2_ and AgBr/bCNTs/TiO_2_ by the two-steps method.

**Figure 2:**
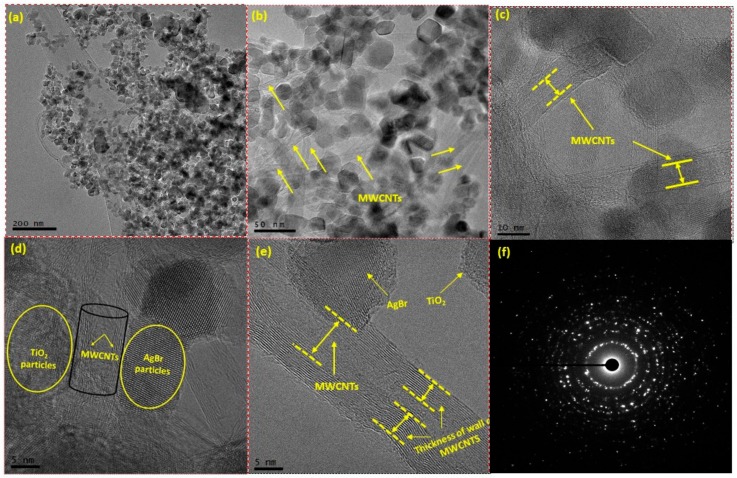
Transmission electron microscope (TEM) images of AgBr/bCNTs/TiO_2_ at different resolutions (**a**) 200 nm; (**b**) 50 nm; (**c**) 10 nm; (**d**) 5 nm; (**e**) 5 nm and (**f**) SAED pattern.

Morphology of AgBr/bCNTs/TiO_2_ was examined by TEM as shown in Figure 2. Uniform distribution of different particles without significant agglomeration is evident in Figure 2a–c. Uniform dispersion of particles supported and endorsed the importance of functionalization and formation of the composite. Figure 2d,e demonstrate higher magnification images showing the unvarying distribution of TiO_2_, AgBr and multi-wall carbon nanotubes. Multiwall structure and thickness of each carbon nanotubes could be clearly observed. Deposition of TiO_2_ and AgBr particles on the walls of CNTs can also be observed, which shows the strong integration of particles with CNTs. The strong bonding among particles would help in efficient charge separation across Z-scheme assembly of the photocatalysts and consequently higher H_2_ production. Furthermore, Figure 2f shows selected area electron diffraction (SAED) image of AgBr/bCNTs/TiO_2_ having several bright spots circles which shows the various crystalline planes of AgBr/bCNTs/TiO_2_. Additionally, TEM images showed that CNTs were uniformly dispersed and no carbon peak in the XRD analysis was observed which was due to the lower amount and even dispersion of CNTs.

**Figure 3:**
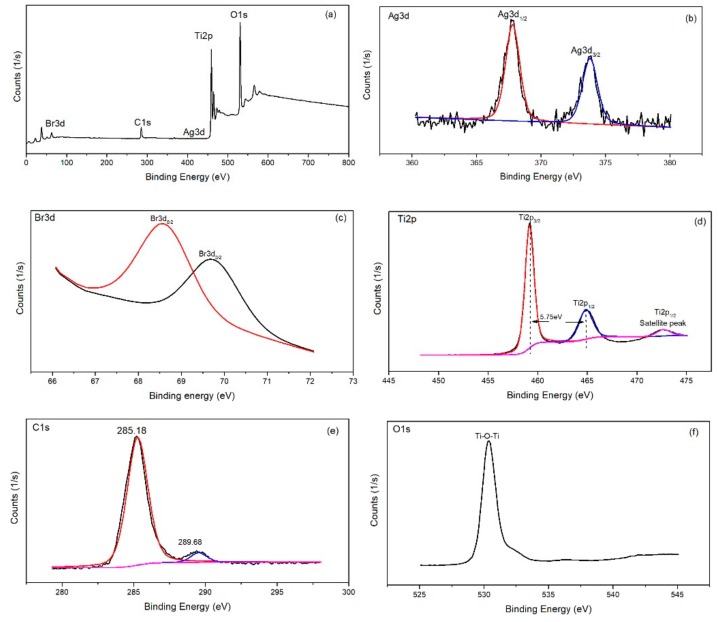
X-ray photoelectron spectra (XPS) analysis of AgBr/bCNTs/TiO_2_ (**a**) Survey spectra; (**b**) Ag3d; (c) Br3d; (**d**) Ti2p; (**e**) C1s and (**f**) O1s.

Surface chemical composition of AgBr/bCNTs/TiO_2_ was investigated through XPS spectroscopy as shown in Figure 3. Survey spectra of AgBr/bCNTs/TiO_2_ exhibited various peaks for silver (Ag), titanium (Ti), bromide (Br), carbon (C) and oxygen (O) as shown in Figure 3a and deconvoluted spectra of individual elements were further plotted to analyse the chemical species in depth as in Figure 3a–d. The Ag spectra presented two major peaks at 367.78 eV and 373.88 eV predicting the Ag3d_1/2_ and Ag3d_3/2_ matching to Ag^+1^ [18]. Valency of Ag was additionally confirmed from the spectra of Br which has peaks at 68.58 and 69.68 eV of Br3d_5/2_ and Br3d_3/2_, corresponding to Br^−1^ in AgBr [19]. The Ti2p spectra has three different peaks at 459.28, 464.98 and 472.68 eV corresponding to Ti^4+^ of Ti2p_3/2_ and Ti 2p_1/2_, respectively [20]. Similarly, C1s spectra produced two peaks at 285.18 and 289.58 eV, respectively. The peaks at 289.58 eV represented the chemical bonding of carbon with oxygen in the form of C–O and C–O–C functional groups. On the other hand, an intense peak at 285.18 eV indicated the sp^2^ characteristics of carbon nanotubes corresponding to C=C, C–C, and C–H bonding [21]. Importantly, the bigger area of peak at 285.18 eV showed that functional groups from the surface of CNTs were eliminated during heat treatment and calcination process. Likewise, O1s spectra of AgBr/bCNTs/TiO_2_ demonstrated the main peak at 530.38 indicating the Ti–O–Ti, and Ti–O–C bonding [22]. Therefore, findings showed the strong bonding of AgBr, CNTs and TiO_2_.

**Figure 4:**
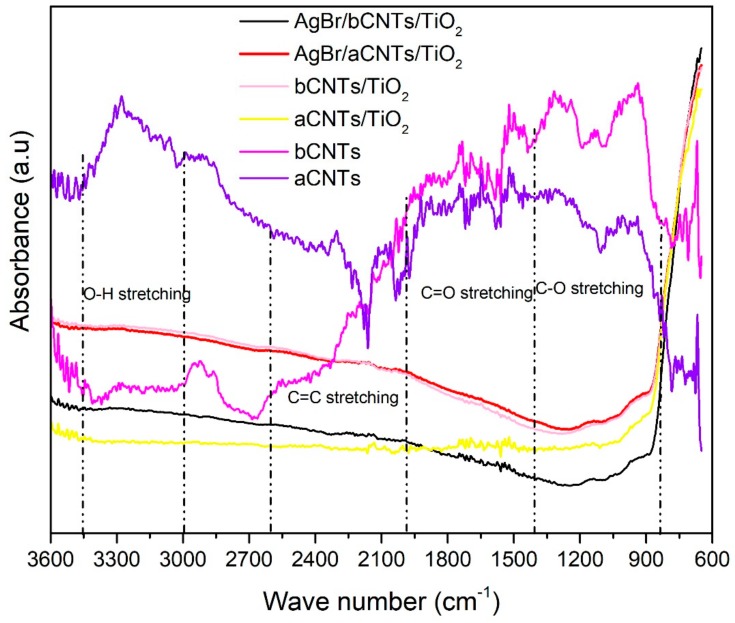
Fourier transform infrared (FTIR) spectra of aCNTs, bCNTs, aCNTs/TiO_2_, bCNTs/TiO_2_, AgBr/aCNTs/TiO_2_ and AgBr/bCNTs/TiO_2_ photocatalysts.

Figure 4 shows the FTIR spectra of aCNTs, bCNTs, aCNTs/TiO_2_, bCNTs/TiO_2_, AgBr/aCNTs/TiO_2_, AgBr/bCNTs/TiO_2_. Spectra have several peaks at different region corresponding to various surface moieties that helped in the development of effective bonding in final composites. In aCNTs and bCNTs, peaks at 3300 cm^−1^ correspond to the OH functional groups. However, intensities of peaks are higher in aCNTs compared to bCNTs. Peaks appearing at 2923 cm^−1^ in bCNTs, and 2890 cm^−1^ in aCNTs represent the –CH stretching. Peaks at 2302 cm^−1^ in aCNTs and peaks at 2183 cm^−1^ in bCNTs showed the COOH bonding [23]. Intensity of COOH bonding is higher in bCNTs compared to aCNTs/TiO_2_. Similarly, bCNTs exhibited peaks at 1493, 1307, 1132 and 943 cm^−1^ in the C=C and –COH groups, whereas peaks were observed at 1556 and 1278 as well 1005 cm^−1^ for C=C and –COH groups in aCNTs [23]. Intensities of these groups are obviously higher in bCNTs which shows that it would help in more effective bonding in composites. In rest of the samples including aCNTs/TiO_2_ and bCNTs/TiO_2_, AgBr/bCNTs/TiO_2_ and AgBr/bCNTs/TiO_2_ most of peaks disappeared because of thermal treatment during the synthesis at high temperature. Only dominant peaks near the 600 cm^−1^ was due to the Ti–O–Ti bonding.

**Figure 5:**
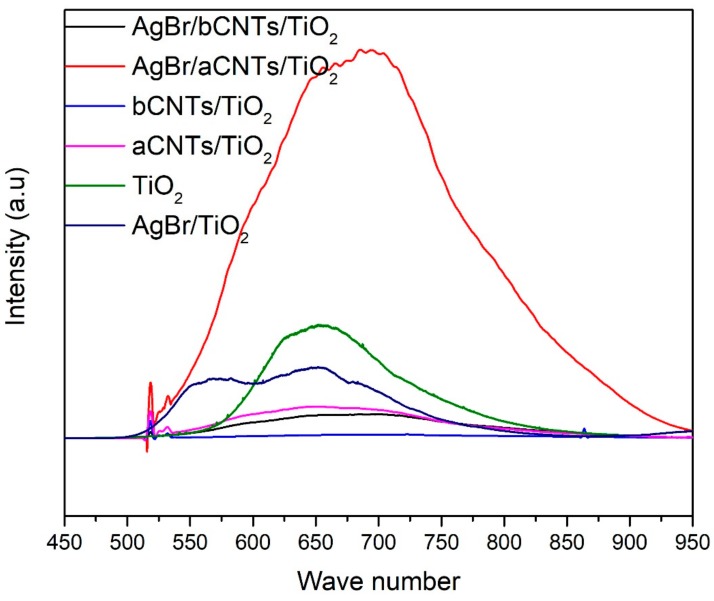
PL spectra of TiO_2_, AgBr/TiO_2_, aCNTs/TiO_2_, bCNTs/TiO_2_, AgBr/aCNTs/TiO_2_ and AgBr/bCNTs/TiO_2_ photocatalysts.

Photogenerated charge carriers trapping, separation, transportation and recombination was estimated by PL spectroscopy as shown in Figure 5. It is obvious that charge recombination intensity of AgBr/aCNTs/TiO_2_ was higher than other photocatalysts owing to the poor bonding between the two semiconductors. The luminance intensity of TiO_2_ compared to aCNTs/TiO_2_, bCNTs/TiO_2_ and AgBr/bCNTs/TiO_2_ was very high because of lower charge separation efficiency. The charge recombination intensity of AgBr/TiO_2_ (8978 a.u) was lower compared to TiO_2_ (14,541 a.u). This shows the better separation of charges in direct Z-scheme assembly of AgBr/TiO_2_ which would be helpful for higher photocatalytic H_2_ production. In addition, aCNTs/TiO_2_, bCNTs/TiO_2_, and AgBr/bCNTs/TiO_2_ exhibited significantly lower intensities compared to that of aCNTs/TiO_2_, AgBr/aCNTs/TiO_2_, AgBr/TiO_2_ and TiO_2_. Remarkable reduced charge recombination intensity indicated the important role of carbon nanotubes as an electron mediator in AgBr/bCNTs/TiO_2_ photocatalysts [24]. Thus, improved charge separation was ascribed to multiwall carbon nanotubes as a solid-state electron mediator in AgBr/TiO_2_.

**Figure 6:**
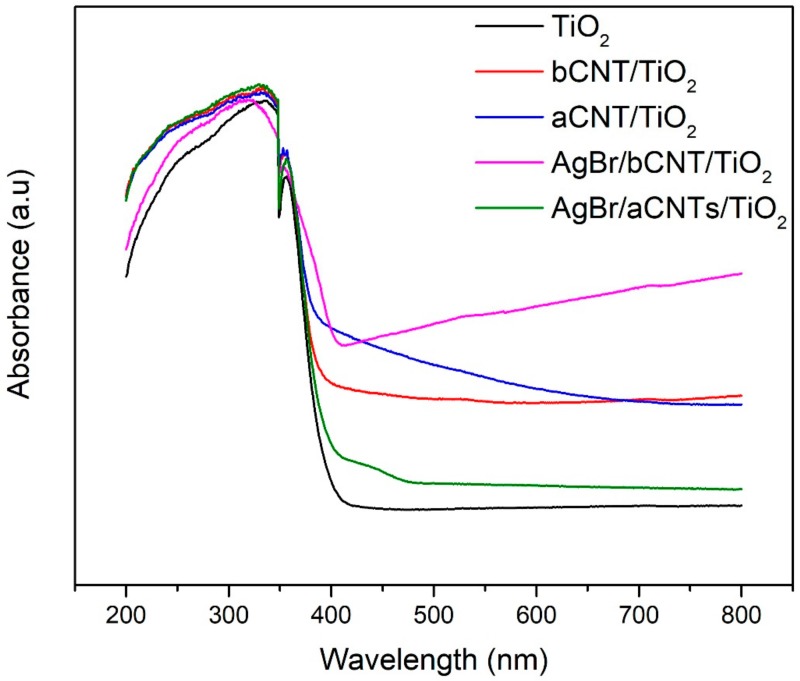
UV-vis spectra TiO_2_, aCNTs/TiO_2_, bCNTs/TiO_2_, AgBr/aCNTs/TiO_2_ and AgBr/bCNTs/TiO_2_ photocatalysts.

The optical behaviour of photocatalyst shows the threshold wavelength of light photons required to initiate the photoreaction. Figure 6 shows the light absorption profile of photocatalysts examined by UV-vis spectrophotometer. All the photocatalysts showed higher absorption in the UV light spectrum owing to the transition of energized electrons from O_2p_ to Ti_3d_ of TiO_2_ [25]. The aCNTs/TiO_2_ and bCNTs/TiO_2_ exhibited little extended light absorption edges compared to TiO_2_ and also higher absorption coefficient. The AgBr/bCNTs/TiO_2_ and AgBr/aCNTs/TiO_2_ exhibited stretched light absorption in the visible light region as compared to bCNTs/TiO_2_ and aCNTs/TiO_2_ and TiO_2_ showing that optical response was greatly enhanced. The light absorption edge of AgBr/bCNTs/TiO_2_ is more stretched into the visible light region compared to the AgBr/aCNTs/TiO_2_ which makes the former more efficient compared to the latter. This will help to produce greater number of photoelectrons and eventually yield of H_2_ production. Moreover, the indirect bandgap energy of AgBr is 2.60 eV which helped to improve the optical response and excitation of photogenerated electrons from valence band (VB) to the conduction band (CB) in AgBr/CNTs/TiO_2_ [26,27]. Thus, improved UV-Vis spectrum of AgBr/bCNTs/TiO_2_ can be regarded as a superposition of AgBr and bCNTs/TiO_2_ optical absorption spectra.

**Figure 7:**
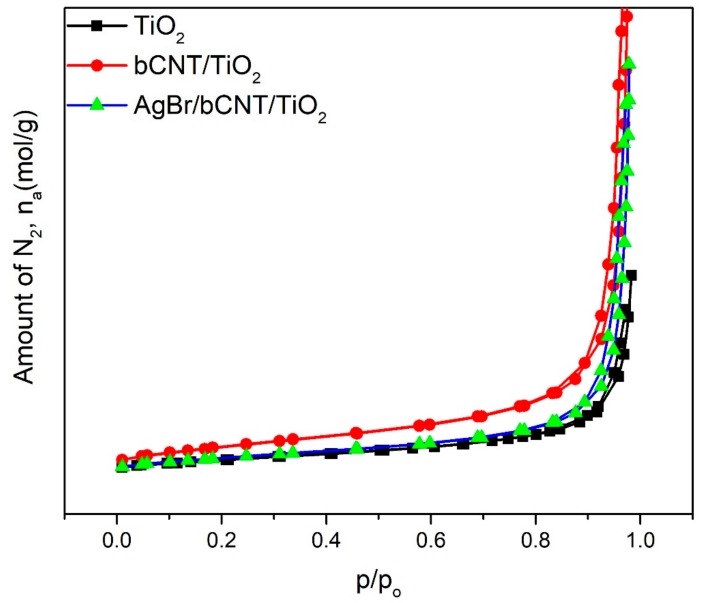
N_2_ adsorption and desorption isotherms of TiO_2_, bCNTs/TiO_2_ and AgBr/bCNTs/TiO_2_ photocatalysts.

Surface areas of TiO_2_, bCNTs/TiO_2_ and AgBr/bCNTs/TiO_2_ were estimated by BELSORP-mini as shown in Figure 7. The N_2_ adsorption and desorption isotherms of TiO_2_, bCNTs/TiO_2_ and AgBr/bCNTs/TiO_2_ resembled type IV isotherm of IUPAC having hysteresis loop in the range 0.8–1 of P/P_o_ and designated the mesoporous structures of photocatalysts. The surface area of TiO_2_ was 45 m^2^ which was increased to 49 m^2^ because of the addition of bCNTs. However, the surface area of final composite, AgBr/bCNTs/TiO_2_, was 47 which was lower than bCNTs/TiO_2_. This decrease in the area was due to the lower surface area of AgBr, and small agglomeration of particles [28].

**Figure 8:**
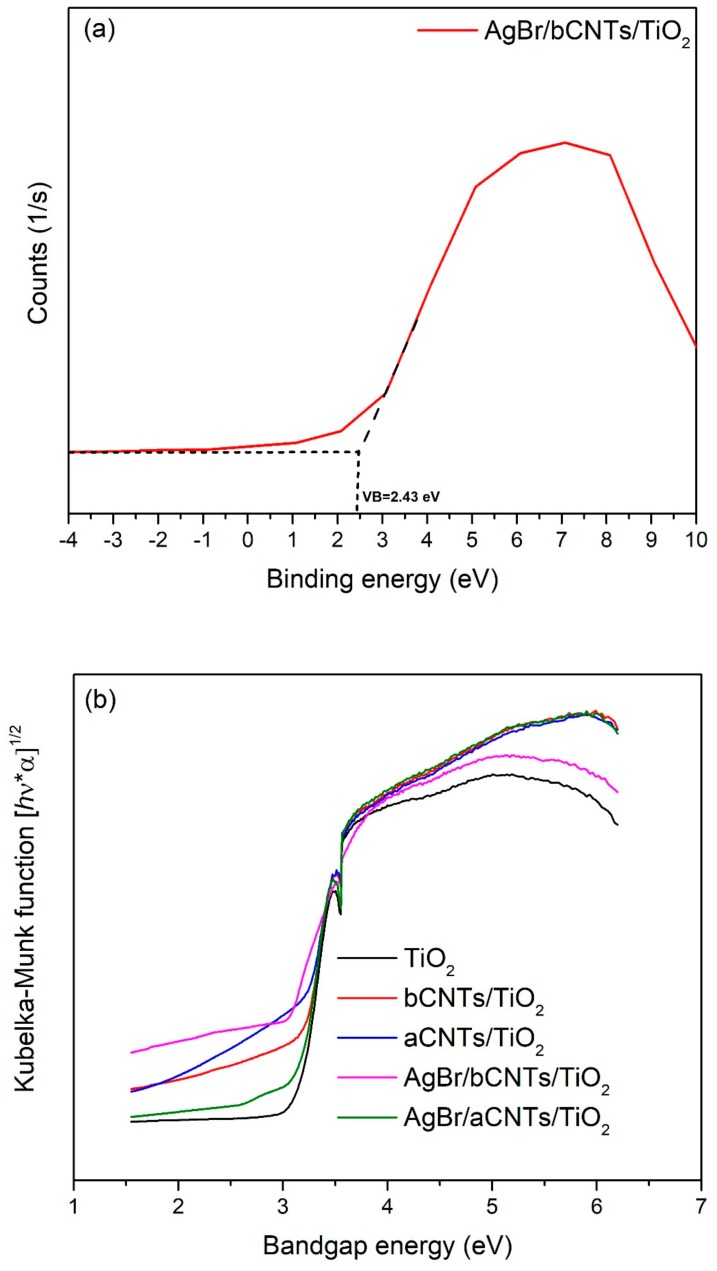
(**a**) VB edge of AgBr/CNTs/TiO_2_. (**b**) Bandgap energy of TiO_2_, aCNTs/TiO_2_ bCNTs/TiO_2_, AgBr/aCNTs/TiO_2_ and AgBr/bCNTs/TiO_2_ photocatalysts.

Further, bandgap structure of AgBr/bCNTs/TiO_2_ photocatalyst were investigated from XPS spectra and Tauc plot of modified Kubelka–Munk function. To measure VB, XPS data were standardized with reference to carbon C1s position at 284.6 eV and were plotted as shown in Figure 8a. The value of VB was measured from the intersection of a tangent line drawn from the curve and straight lines from the initial point of curves. The measured value of VB was 2.42 eV, whereas reported VB potential of AgBr and TiO_2_ are 2.30 eV and 2.7 eV, respectively [29]. Modified VB value indicated the stronger bonding among TiO_2_, CNTs and AgBr. Furthermore, Figure 8b shows the Tauc plot of modified Kubelka–Munk (KM) function vs. photoenergy/bandgap to estimate the bandgap energies of different photocatalysts. Bandgap energy of AgBr/bCNTs/TiO_2_ was 2.62 eV which is lower than TiO_2_ (3.2 eV). Lower bandgap energy of photocatalyst designated the higher expected light absorption and photocatalytic efficiency. The value of CB was calculated using Equation (1) given as follows.

(1) $$E_{\mathrm{VB}}={\;E}_{\mathrm{CB}}+{\;E}_{g}$$

where ${\;E}_{g}$, $E_{\mathrm{VB}}$ and $E_{\mathrm{CB}}$ are the bandgap energy, VB and CB potentials of semiconductors. The $E_{CB}$ of AgBr/bCNTs/TiO_2_ was found to be −0.2 eV, whereas stated $E_{\mathrm{CB}}$ of AgBr and TiO_2_ are −0.30 and −0.5 eV, respectively. Altered CB position of AgBr/bCNTs/TiO_2_ was ascribed to the bCNTs and AgBr.

### 3.2. Photocatalytic H_2_ Production

**Figure 9:**
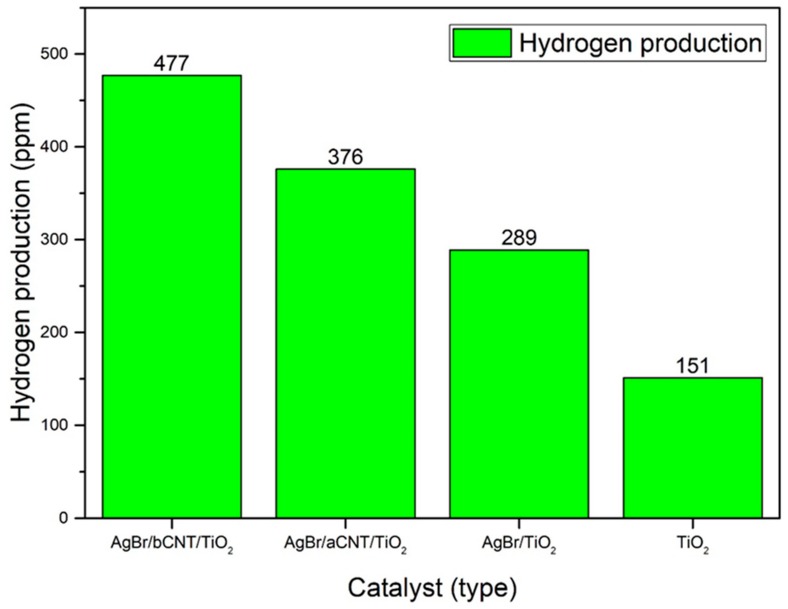
Photocatalytic H_2_ production over various photocatalysts.

The photocatalytic performance of all the composites was assessed for H_2_ generation from water using methanol as hole scavenger under visible light irradiations. Production of H_2_ using different photocatalysts is shown in Figure 9. Pure TiO_2_ produced 151 ppm of H_2_, which was lower than other photocatalysts owing to its large bandgap energy and severe charge recombination. The AgBr/TiO_2_ photocatalyst exhibited 289 ppm of H_2_ almost twice than benchmark TiO_2_. The AgBr/bCNTs/TiO_2_ and AgBr/aCNTs/TiO_2_ photocatalysts exhibited higher yield of 477 ppm and 376 ppm of H_2_ which were greater than TiO_2_. The significant increase in yield of the H_2_ was due to lower bandgap energy and efficient charge recombination. Also, incorporation of CNTs boosted the amount of H_2_ production owing to higher surface area, electrical, and optical properties. Therefore, enhanced efficiency of AgBr/bCNTs/TiO_2_ could be credited to efficient charge separation, light absorption, and indirect Z-scheme assembly developed between AgBr and TiO_2_ through bridge of CNTs.

Evidently, the optical behaviour of AgBr/bCNTs/TiO_2_ was far better than TiO_2_ which played a pivotal role in the enhancement of photocatalytic activity under visible light irradiations. Bandgap energy of AgBr/bCNTs/TiO_2_ was 2.62 eV, lower than pure TiO_2_ because of the incorporation of CNTs and AgBr. The modified optical response enabled the composites to work efficiently under visible light irradiations and led to 477 ppm of H_2_ production. Moreover, the conduction band position of composite was −0.2 eV, which favoured the water splitting. It is due to the fact that the reduction potential for H_2_ is 0 eV, whereas CB of AgBr/bCNTs/TiO_2_ was −0.20 eV, higher than overall reduction potential required for water splitting and sufficient to produce the H_2_ [30]. In addition, the dominant factor for the enhancement in H_2_ production was efficient charge separation and transportation capability of photocatalyst. Charge recombination intensity of composites was 89 times lower than simple TiO_2_ owing to the development of Z-scheme heterojunction. Incorporation of CNTs increased the surface area and enhanced the charge separation because of the excellent electrical conductivity [24]. Separated electrons and holes on the surface of photocatalyst carried out redox reaction and exhibited a remarkable improvement in H_2_ production. The AgBr/aCNTs/TiO_2_ charge separation was very poor compared to the AgBr/bCNTs/TiO_2_ and led to the lower H_2_ production. Further, the dual role of CNTs was very important owing to the generation of greater active sites because of the greater area, surface functional groups and higher diffusion capability. Diffusion of charge through tubular structure lowered the charge recombination and led to good separation and transportation [24,31,32].

Moreover, as an electron mediator, the activity of acid functionalized CNTs was quite different from the basic one. The H_2_ production rate of acid-functionalised aCNTs-based photocatalyst was 376 ppm whereas base-functionalised bCNTs-based photocatalyst exhibited 477 ppm of H_2_, which was 1.27 times higher than the former. The difference of yield was attributed to functional groups attached on the surface of bCNTs. The functional moieties on the bCNTs surface affected the interfacial bonding between AgBr/TiO_2_ and bCNTs. Strength of bonding was responsible for charge recombination [33]. Higher efficiency of the base-functionalised CNTs-based composites showed that there was uniform dispersion and strong bonding among CNTs, TiO_2_ and AgBr which led to efficient charge separation and transportation. Quickly migrated electrons and holes attacked the H^+^ and reduced it into H_2_. On the other hand, the comparatively lower yield of H_2_ over aCNTs-based photocatalyst revealed that AgBr and TiO_2_ interfacial bonding strength was weaker than the former which led to relatively lower production of solar fuel. Also, functional groups present on the surface of the photocatalysts help in redox reaction and intermediate formation which further enhance the H_2_ production. In addition to the interfacial bonding strength, basic and acidic moieties on the surface of photocatalyst affected the dispersibility of the photocatalyst. It was visualised that dispersion of bCNTs-based photocatalyst was better and uniform as compared to aCNTs-based photocatalyst. Uniform dispersion of active photocatalyst particles enable the efficient harnessing of visible light irradiations and led to higher H_2_ production. This can be further better explained by considering the affinity of AgBr particles. The AgBr particle is highly soluble in basic solution. The solubility of AgBr particles strongly affects the deposition process. When basic solvents were attached on the surface of the bCNTs, they promoted the interaction of depositing particles of AgBr on the surface of bCNTs. Uniform distribution and strong interaction of AgBr and bCNTs fortified the effective bonding of light-sensitive element with electron mediator. In the case of acid solvents, which is hydrophobic to the AgBr particles lead to poor dispersion and weaker interfacial bonding and eventually lower comparative efficiency of the photocatalyst. The difference in the behaviour of both the composites was due to the different nature of the functional groups present on the surface. Therefore, surface chemical species on electron mediator strongly influence the interfacial bonding and hence, yield of solar fuels [33,34,35].

**Figure 10:**
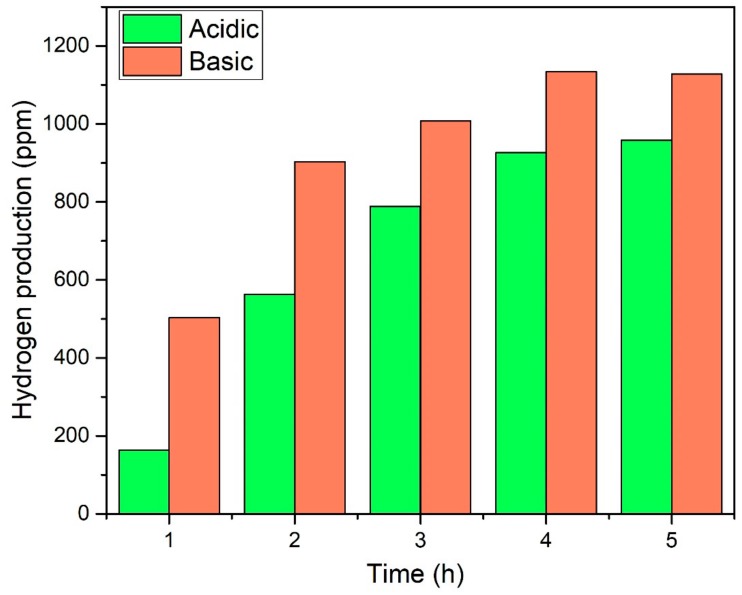
Effect of time on yield of H_2_ over AgBr/aCNTs/TiO_2_ and AgBr/bCNTs/TiO_2._

**Figure 11:**
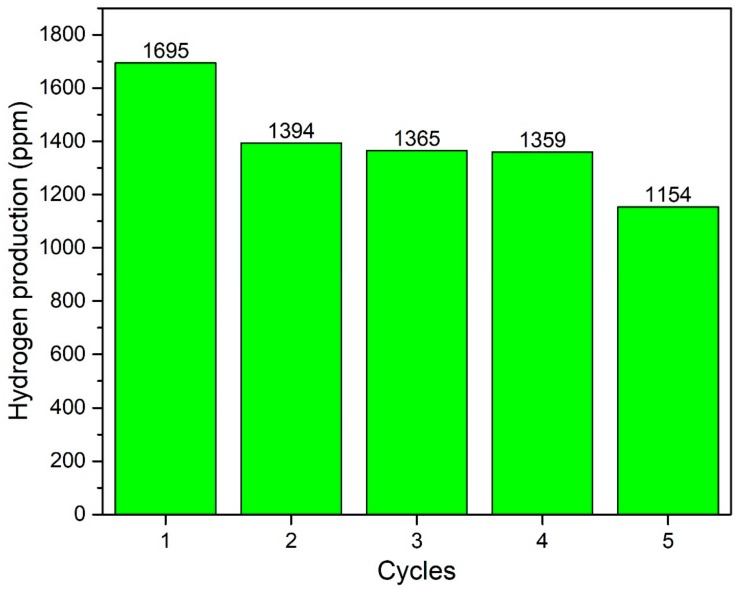
Stability analysis of AgBr/bCNTs/TiO_2_ for H_2_.

In addition, effect of time was observed for the acid- and base-functionalised CNTs-based composites by performing the reaction for 5 h continuously as plotted in Figure 10. Both the photocatalysts demonstrated smooth production of H_2_. The yield was initially increased with time and then become almost constant. Further, recyclability analysis was performed for consecutive five cycles and photocatalyst exhibited almost the same yield of H_2_ without losing activity as shown in Figure 11. Notable stability of photocatalyst was due to good interfacial bonding, generation and transportation of the photogenerated electrons, resulting in enhanced spatial charge separation [21,36]. The AgBr is highly sensitive to light and immediately decomposes to Ag and Br. Reason of decomposition could be the inability of generated charge carriers to move to other elements because of lower electrical conductivity or poor bonding. The stable activity was observed for five consecutive cycles owing to the smooth transfer of electrons across the electronic interface between AgBr and TiO_2_ through bCNTs. Similarly, Xin et al. [37] reported that the activity of Ag/AgBr/GdVO_4_ was stable without any significant loss for five consecutive cycles. Likewise, Xu et al. [38] used the AgBr/AgIn(MoO_4_)_2_ photocatalysts for the degradation of dyes and observed stable and smooth performance for six cycles. Thus, the stable photocatalytic performance of AgBr/bCNTs/TiO_2_ was credited to the bCNTs as an electron mediator and stronger bonding because of the surface functional which enabled efficient separation of charges and reduced the recombination intensity. In addition, comparison was made with other studies reported in literature as shown in Table 1. It shows that photocatalytic H_2_ in current study is comparable and even higher than many photocatalysts. However, true comparison cannot be developed till process parameters and reactor geometry are identical.

### 3.3. Mechanism of H_2_ Generation on AgBr/bCNTs/TiO_2_

**Figure 12:**
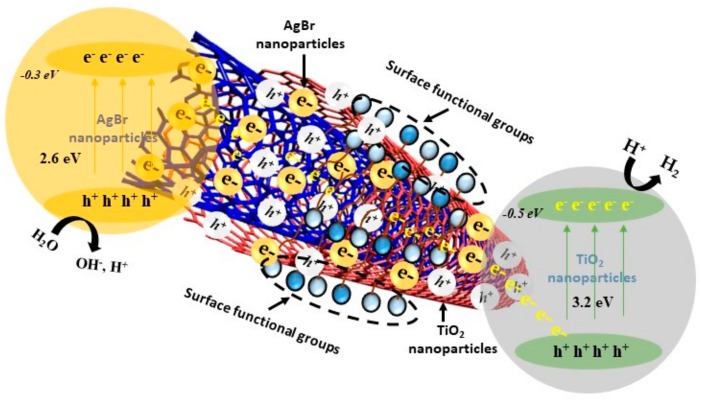
Mechanism of H_2_ production over AgBr/bCNTs/TiO_2_ photocatalyst.

Water splitting involves complex redox reaction on the surface of photocatalyst and therefore it is crucial to understand the mechanism of H_2_ production using photocatalysis. Figure 12 illustrates the mechanism of H_2_ production over AgBr/bCNTs/TiO_2_. It could be seen that when light irradiations strike the surface of photocatalyst, electrons started moving from the VB to CB of AgBr. Photogenerated electrons jumped to the carbon nanotubes which were acting as a bridge in transferring the charge carriers from AgBr to TiO_2_. Separated electrons take part into redox reaction thereby reducing the H^+^ into H_2_ and holes left on AgBr attacked the adsorbed H_2_O molecules to oxidize it to H^+^ and O_2_. Further, CB potentials of TiO_2_ and AgBr are −0.5 and −0.3 eV, respectively, which determine the mechanism of transfer of electrons and hence redox reaction [7,46,47]. In AgBr/bCNTs/TiO_2_, AgBr is a light-sensitive element and emits electrons under visible light irradiations. These electrons are transferred to the TiO_2_ for H_2_ production else could recombine on the surface of AgBr leading to its decomposition and loss of activity. The CB of TiO_2_ is higher than AgBr, therefore, the excited electrons move to the VB of TiO_2_ only, resulting in the formation of Z-scheme assembly of AgBr/TiO_2_. As observed in PL spectra and H_2_ production results, the efficiency of AgBr/TiO_2_ was better than pure TiO_2_ but considerably lower than AgBr/bCNTs/TiO_2_. These results indicated that the presence of CNTs was very important to enhance the efficiency of Z-scheme AgBr/TiO_2_ because of its outstanding electrical conductivity as it reduced the charge recombination and also enhance the light absorption capability. Photocatalytic reduction of H^+^ into H_2_ depends upon the CB potential of a photocatalyst. For AgBr/bCNTs/TiO_2_, overall CB was −0.2 and VB was 2.42 which was sufficient to convert H_2_O into H_2_ and O_2_. Further, the standard thermodynamics redox potential of H_2_ is 0 eV, while theoretically redox potential of O_2_ is 1.23 eV [48]. Similarly, individual CB positions of AgBr and TiO_2_ are −0.30 and 0.5 eV, respectively [7,46,47]. Therefore, indirect Z-scheme assembly of AgBr/bCNTs/TiO_2_ was an efficient photocatalyst for photocatalytic H_2_ production form H_2_O.

## 4. Conclusions

Carbon nanotubes-incorporated Z-scheme assembly of AgBr/TiO_2_ photocatalysts were successfully prepared by facile deposition and reflux method. The photocatalysts exhibited great potential for H_2_ production with maximum yield of 477 ppm of H_2_. The performance of AgBr/bCNTs/TiO_2_ was higher than AgBr/aCNTs/TiO_2_, AgBr/TiO_2_ and TiO_2_, respectively. The charge recombination intensity of AgBr/bCNTs/TiO_2_ was 89 times lower than TiO_2_ and bandgap energy was 2.62 eV showing that properties of TiO_2_ were greatly improved. Further, the photocatalyst exhibited stability for consecutive five cycles showing its potential for continuous H_2_ production under visible light irradiations. Remarkable activity was ascribed to strong interfacial bonding, high surface area, reduced charge recombination and improved visible light response because of the incorporation of CNTs and surface functional groups. Thus, this study would be helpful to develop new Z-scheme photocatalyst with enhanced efficiency for various photocatalytic applications.

## Figures and Tables

**Table 1:** Comparison of yield of H_2_ production over various photocatalysts.

Photocatalyst	Methodology	Process Parameters	Yield of H_2_	Reference
CdS nanoflower/rutile-TiO_2_ nanorod	Hydrothermal method	Photoelectrochemical cell, Solar simulator, 100 mW/cm^2^	336 μmol/h.	[39]
Ag-TiO_2_ nanorods	Hydrothermal and photodeposition method	Pyrex glass tube, 125 W Hg arc lamp	105 μmol	[40]
AgBr/rGO/TiO_2_	Reflux and thermal treatment	Pyrex glass reactor, 35 W Xe lamp,	2025 ppm	[5]
Pt/TiO_2_	Sol–gel method	Pyrex glass reactor, 400 W mercury lamp,	1023.71 μmol/g/h	[41]
Plasmonic Cu/TiO_2_	Sol-immobilisation method	Double-wall quartz glass reactor, 1000 W Xe-Lamp	160 μmol/g/h	[42]
N doped TiO_2_	Anodisation method	CEL-SPH2N system, 300 Xe lamp	15.1 μmol	[43]
TiO_2_@rGO@Au	Deposition	Photoelectrochemical cell, 300 W Xe light	17.8 μmol/cm^2^	[44]
TiO_2_/3D graphene	Hydrothermal method	Pyrex vessel photoreactor, 200 W Xe arc lamp	1205 μmol/g/h	[45]
AgBr/bCNTs/TiO_2_	Deposition and reflux method	Pyrex glass reactor, 35 W Xe lamp,	477 ppm	This study
